# NIR Stimulus-Responsive PdPt Bimetallic Nanoparticles for Drug Delivery and Chemo-Photothermal Therapy

**DOI:** 10.3390/pharmaceutics12070675

**Published:** 2020-07-17

**Authors:** Chun Chu, Zhihong Bao, Meng Sun, Xiaowei Wang, Hongyan Zhang, Weiguo Chen, Yang Sui, Ji Li, Yuanyuan Zhuang, Dongkai Wang

**Affiliations:** School of Pharmacy, Shenyang Pharmaceutical University, Shenyang 110016, China; chuchunpharmacy@163.com (C.C.); baozhihong1982@163.com (Z.B.); sunmeng971012@163.com (M.S.); wxw15940446926@163.com (X.W.); 13188235157@163.com (H.Z.); cwghello@foxmail.com (W.C.); suiyang0309@gmail.com (Y.S.); syphuliji@hotmail.com (J.L.); zhuangyuanyuan1998@163.com (Y.Z.);

**Keywords:** PdPt bimetallic nanoparticles, drug delivery, chemo-photothermal therapy, anti-tumor effect

## Abstract

The combination of chemotherapy and phototherapy has attracted increasing attention for cancer treatment in recent years. In the current study, porous PdPt bimetallic nanoparticles (NPs) were synthesized and used as delivery carriers for the anti-cancer drug doxorubicin (DOX). DOX@PdPt NPs were modified with thiol functionalized hyaluronic acid (HA-SH) to generate DOX@PdPt@HA NPs with an average size of 105.2 ± 6.7 nm. Characterization and in vivo and in vitro assessment of anti-tumor effects of DOX@PdPt@HA NPs were further performed. The prepared DOX@PdPt@HA NPs presented a high photothermal conversion efficiency of 49.1% under the irradiation of a single 808 nm near-infrared (NIR) laser. Moreover, NIR laser irradiation-induced photothermal effect triggered the release of DOX from DOX@PdPt@HA NPs. The combined chemo-photothermal treatment of NIR-irradiated DOX@PdPt@HA NPs exerted a stronger inhibitory effect on cell viability than that of DOX or NIR-irradiated PdPt@HA NPs in mouse mammary carcinoma 4T1 cells in vitro. Further, the in vivo combination therapy, which used NIR-irradiated DOX@PdPt@HA NPs in a mouse tumor model established by subcutaneous inoculation of 4T1 cells, was demonstrated to achieve a remarkable tumor-growth inhibition in comparison with chemotherapy or photothermal therapy alone. Results of immunohistochemical staining for caspase-3 and Ki-67 indicated the increased apoptosis and decreased proliferation of tumor cells contributed to the anti-tumor effect of chemo-photothermal treatment. In addition, DOX@PdPt@HA NPs induced negligible toxicity in vivo. Hence, the developed nanoplatform demonstrates great potential for applications in photothermal therapy, drug delivery and controlled release.

## 1. Introduction

Chemotherapy has contributed greatly to reduce cancer mortality but usually accompanies serious side effects and lacks specificity toward tumor cells [1,2]. The combination of chemotherapy and phototherapy, which could be delivered by nanoscale carriers for precise drug delivery, was considered an attractive approach to reducing side effects and enhancing therapeutic efficacy in cancer treatment in recent years [3,4,5].

Photothermal therapy (PTT) is one of the primary types of phototherapy methods that employs the heat generated from the absorbed optical energy by photothermal agents to ablate tumor cells [6,7]. Numerous metals including Au, Pd and Pt have been reported to possess near-infrared (NIR) laser-induced photothermal effects in cancer therapy [8,9,10,11,12]. To realize combined chemo-photothermal therapy, anti-cancer drugs were loaded into the nanoparticles (NPs) containing photothermal agents [13,14]. Apparently, the strategies to prepare NPs with both photothermal property and drug-loading capacity are anticipated for the advantages of simplifying the synthesis process [15].

Plenty of PdPt heteronanostructures, including polyhedral shapes nanocrystals [16,17,18], nanoplates [19] and porous nanodendrites, [20,21] have been synthesized to improve their electrocatalytic activity towards oxygen reduction reactions, methanol oxidation reactions and ethanol oxidation reactions. Importantly, PdPt nanoplates were reported to exert a photothermal effect in Pd@Pt-PEG-Ce6 nanocomposite that facilitate permeation of photosensitizer Ce6 into breast cancer cells with enhanced efficacy of photodynamic therapy [19]. However, the 2D structure of PdPt nanoplates limits its utilization as a drug carrier. The porous structure provides NPs with high loading capacities for anti-cancer drugs [15]. Fang et al. synthesized porous nanostructures of PdPt through an easily operated synthetic routine with the regulation of reduction kinetic of the metal precursors [21], while the possibility of porous PdPt NPs used as efficient vehicles for drug delivery and photothermal agents remains to be elucidated.

Hyaluronic acid (HA) is a natural high molecular weight glycosaminoglycan found in the extracellular matrix and synovial fluids of the body [22,23,24]. Cluster of differentiation 44 (CD44), a cell surface glycoprotein expressed on multiple tumor cells, acts as a principal transmembrane adhesion receptor for HA [25,26,27]. The specific binding of HA with CD44 overexpressed solid tumors provides HA-coating NPs with actively targeting capability. Hyaluronidase that is abundant in tumor regions further degrades HA to realize tumor-responsive drug release [23,28,29]. Moreover, HA also has high thermal stability [30], the characteristic of HA renders it an excellent candidate to cover NPs for PTT. 

**Figure 1:**
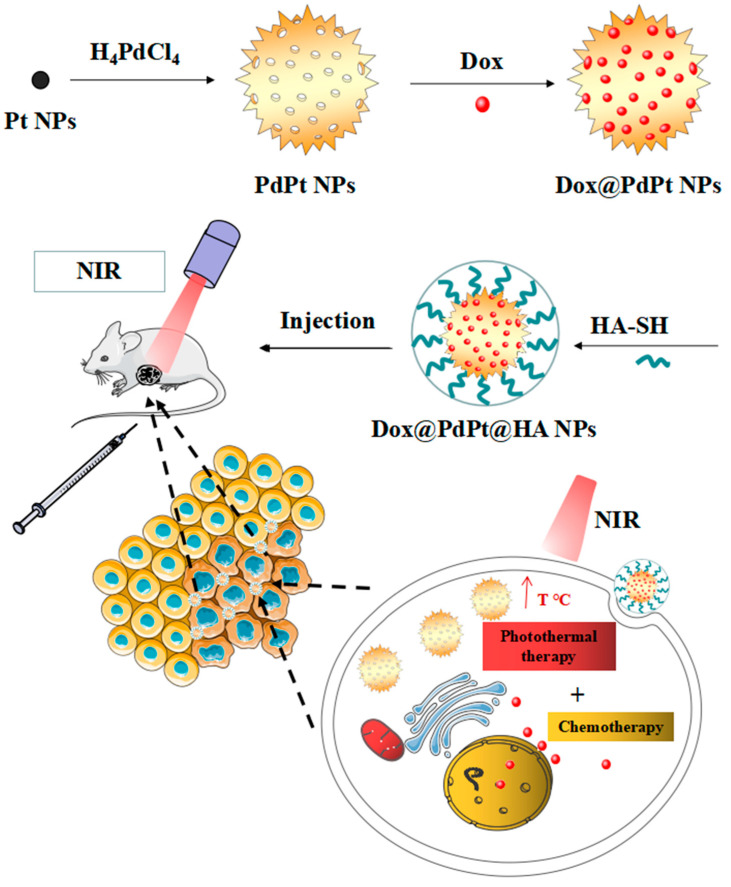
Schematic illustration of preparation of DOX@PdPt@HA nanoparticles (NPs) and near infrared (NIR) laser irradiation-induced hyperthermia triggered doxorubicin (DOX) release for chemo-photothermal therapy. The figures were produced using Servier Medical Art, which is licensed under a Creative Commons Attribution 3.0 Unported License.

In the present study, the porous PdPt NPs were synthesized and loaded with anti-cancer drug doxorubicin (DOX). HA was used for further DOX@PdPt NPs coating to generate DOX@PdPt@HA NPs (Figure 1). Photothermal conversion efficiency, DOX loading capacity and NIR illumination-responsive DOX release were evaluated. The anti-cancer activity of DOX@PdPt@HA NPs in vitro and in vivo was determined. The generated porous PdPt NPs exerted high photothermal conversion efficiency and DOX loading efficacy. Laser-induced hyperthermia was shown to accelerate DOX release from DOX@PdPt@HA NPs. Moreover, chemo-photothermal treatment using NIR-irradiated DOX@PdPt@HA NPs exhibited higher therapeutic efficacy in comparison with stand-alone chemotherapeutic or photothermal treatment. Our findings suggest a promising application of the porous PdPt NPs for anti-tumor drug delivery and PTT.

## 2. Materials and Methods

### 2.1. Materials 

DOX, H_2_PtCl_6_, H_2_PdCl_4_, NaBH_4_, cetyltrimethylammonium chloride (CTAC), ascorbic acid (AA), 1-hydroxybenzotriazole (HOBt), 1-ethyl-3-[3-(dimethylamino)propylcarbodiimide] (EDC), cystamine dihydrochloride, dithiothreitol (DTT) and cystamine dihydrochloride were purchased from Shanghai Aladdin Reagent Company (Shanghai, China). Fetal bovine serum (FBS) was obtained from Clark (Richmond, VA, USA) and Dulbecco’s modified Eagle’s medium (DMEM) was purchased from HyClone (Logan, UT, USA). Annexin V-FITC/propidium iodide (AV/PI) apoptosis detection kit and 3-(4,5-dimethylthiazol-2-yl)-2,5-diphenyltetrazolium bromide (MTT) were obtained from Sigma-Aldrich (St. Louis, MO, USA). Biochemical kits for alanine aminotransferase (ALT) and aspartate aminotransferase (AST) assay were obtained from Wanlei Life Science (Shenyang, China). Hematoxylin-eosin (H&E) staining kit, primary antibodies against Ki-67 and caspase-3, as well as HRP-conjugated secondary antibodies were purchased from Servicebio (Wuhan, China). Anti-fade mounting medium with DAPI was purchased from Beyotime Biotechnology (Shanghai, China).

### 2.2. Synthesis of PdPt NPs 

PdPt NPs were generated based on a protocol previously reported [21]. In brief, the seed solution was formed by adding the ice-cold NaBH_4_ (0.01 M, 600 µL) into H_2_PtCl_6_ (0.01 M, 100 µL) and CTAC (0.1 M, 10 mL) mixture to generate Pt NPs as the seeds. And 200 µL seed solution was then injected into a mixture of H_2_PtCl_6_ (0.01 M, 660 µL), H_2_PdCl_4_ (0.01 M, 120 µL), AA (0.1 M, 320 µL) and CTAC (0.1 M, 40 mL). After aged at 30 °C for 3 days, PdPt NPs were collected by centrifugation at 13,000 rpm/min before characterization. 

### 2.3. Preparation of Thiol Functionalized HA (HA-SH)

HA-SH was synthesized by following a previous report [31]. Briefly, 125 mL HA (MW 10 kDa, 6.25 mmol) was mixed well with EDC (18.75 mmol) and HOBt (18.75 mmol) under constant stirring for 2 h. Then, 18.75 mmol of cystamine dihydrochloride was introduced to generate cystamine conjugated HA. After overnight incubation, the dialysis membrane tubing (MW cutoff of 3500 Da) was used to remove unreacted coupling agents. DTT (31.25 mmol) was then added to cystamine conjugated HA to cleave the disulfide bond in the conjugated cystamine. HA-SH was lyophilized after two days of dialysis (MW cutoff of 6000 to 8000 Da) and stored at −20 °C. The chemical shift difference in proton nuclear magnetic resonance (^1^H-NMR) spectrum between HA and HA-SH was obtained on a Bruker AVANCE III HD spectrometer operating at 600 MHz (Bruker, Billerica, MA, USA) (Figure S1). 

### 2.4. Preparation of DOX@PdPt@HA 

DOX@PdPt NPs were prepared by dissolving a mixture of 2 mg DOX and 40 µg PdPt NPs in 1 mL PBS buffer (pH 7.0). DOX-loaded PdPt NPs were separated by a 10 min centrifugation step at 13,000 rpm/min and further washed with water three times to remove the unloaded DOX. The unloaded DOX was quantified by using a UV-vis spectrophotometer (Unico Instrument Co. Ltd., Shanghai, China) at 480 nm according to the DOX calibration curve and the loading content was calculated by following the equation [32]

(1) $$\mathrm{Loading}\;\mathrm{content}\;(\%)=\frac{\mathrm{Total}\;\mathrm{DOX}\;\mathrm{added}\;-\;\mathrm{Free}\;\mathrm{DOX}}{\mathrm{Weight}\;\mathrm{of}\;\mathrm{nanocomposite}}\;\times \;100\%.$$

DOX@PdPt@HA NPs were prepared by introducing 50 mg HA-SH to coat DOX-loaded PdPt NPs in 10 mL PBS solution at pH 7.0.

### 2.5. Characterization 

The transmission electron microscopy (TEM) imaging of Pt seed, PdPt and DOX@PdPt@HA NPs was obtained on a Hitachi HT7700 transmission electron microscope (Tokyo, Japan) operating at 120 kV. The size of a minimum of 300 particles per sample was measured to determine the average size of NPs [33]. The composition of PdPt NPs was acquired by Agilent 7700x ICP-MS (Agilent Technologies, Tokyo, Japan). The absorption spectrum was collected by UV-vis-NIR spectrophotometer (Thermo Scientific, Wilmington, DE, USA). Infrared spectroscopic analyses were performed using a Fourier transform infrared (FTIR) spectrometer (IFS55; Bruker, Billerica, MA, USA). ζ-potential and polydispersity index (PDI) were measured on a Malvern ZEN 3600 Zetasizer (Malvern Instruments, Worcestershire, UK).

### 2.6. Measurement of Photothermal Effect 

PdPt, PdPt@HA and DOX@PdPt@HA NPs (20 µg/mL) were irradiated by 808 nm laser (0.9 W/cm^2^, LEO Photonic Co., Ltd. Shenzhen, China) for 10 min and the real-time temperature was recorded and infrared thermographic maps were obtained by the infrared thermal imaging camera (LaserSight, Optris, Germany). The photothermal conversion efficiency (η) of PdPt, PdPt@HA and DOX@PdPt@HA NPs was calculated by following the published equations [34]. 

### 2.7. Release of DOX from DOX@PdPt@HA NPs 

DOX@PdPt@HA NPs solution with or without laser irradiation (808 nm, 0.9 W/cm^2^, 10 min) at the 1, 3, 5, 7 and 9 h time points was dialyzed (MW cutoff of 3500 Da) against PBS, the absorption spectrum of outer solution was collected for quantifying the concentration of released DOX and the same volume of PBS was added to the reservoir to keep a constant volume. 

### 2.8. Cell culture

Mouse mammary carcinoma 4T1 cells (Cell Bank of the Chinese Academy of Sciences, Shanghai, China) and human foreskin fibroblast (HFF; ATCC, Manassas, VA) cells were cultured in DMEM complete medium containing 100 U mL^−1^ penicillin, 100 U mL^−1^ streptomycin and 10% FBS. Both kinds of cells were incubated at 37 °C with 5% CO_2_ in a humidified atmosphere during the study. An inverted microscope (AE31; Motic Electric, Xiamen, China) was used to visualize cell morphology.

### 2.9. MTT Assay 

The 4T1 cells and HFF cells were cultured in 96-well plates (5 × 10^3^ cells per well) with DMEM complete medium. After overnight incubation, the culture medium of HFF cells was removed and replaced by 100 µL fresh DMEM complete medium containing PdPt NPs (10, 20 and 40 µg/mL) or PdPt@HA NPs (10, 20 and 40 µg/mL). The culture medium of 4T1 cells was replaced by fresh DMEM complete medium containing free DOX (2.33, 4.66 and 9.32 μg/mL), PdPt@HA NPs (10, 20 and 40 µg/mL) or DOX@PdPt@HA NPs (containing the equivalent amount of DOX and PdPt@HA NPs). To determine NIR laser-induced cell death, 4T1 cells were incubated with NPs for 1 h, then the cover of the plate was removed and the desired wells were exposed to laser irradiation (808 nm, 0.9 W/cm^2^) for 10 min. After 24 h incubation, the cells were rinsed twice with PBS and incubated with 100 µL MTT solution (0.5 mg/mL) for another 2 h. The cells in the control group were incubated with medium alone. The medium was then removed and 100 µL DMSO was added to each well to dissolve formazan crystals. The optical density (OD) of each sample was measured by a spectrophotometer (F900; Edinburgh Instruments Ltd., Edinburgh, UK) at 570 nm. Cell viability was calculated using the following equation

(2) $$\mathrm{Cell}\;\mathrm{viability}=\frac{{\mathrm{OD}}_{570\mathrm{nm},\;\mathrm{sample}}{\;-\;\mathrm{OD}}_{570\mathrm{nm},\;\mathrm{blank}}}{{\mathrm{OD}}_{570\mathrm{nm},\;\mathrm{control}}{\;-\;\mathrm{OD}}_{570\mathrm{nm},\;\mathrm{blank}}}\;\times \;100\%.\;$$

### 2.10. Cell Death Examined by AV-PI Double Staining 

The 4T1 cells (2 × 10^5^ cells/well) were incubated in 6-well plates overnight. Then, 4T1 cells were stimulated by free DOX (4.66 μg/mL), PdPt@HA NPs (20 µg/mL) or DOX@PdPt@HA NPs (containing the equivalent amount of DOX and PdPt@HA NPs) in 2 mL DMEM complete medium for 1 h to allow the uptake of these reagents and fresh medium was added as control. After NIR laser irradiation (808 nm, 0.9 W/cm^2^, 10 min), cells were cultured for additional 24 h before cell collection by centrifugation. Then, cells were resuspended in 500 μL buffer containing 5 μL Annexin V-FITC and 10 μL PI provided in an AV/PI apoptosis detection kit (Sigma-Aldrich, St. Louis, MO, USA). After 10 min incubation, the percentage of apoptotic4T1 cells was determined by a flow cytometer (Becton Dickinson Biosciences, San Jose, CA, USA). 

### 2.11. CD44-Mediated Delivery of DOX@PdPt@HA NPs

CD44-mediated delivery of DOX@PdPt@HA NPs was examined by following a protocol based on published literature [35]. In brief, 4T1 cells were seeded onto glass slides in 6-well culture plates (5 × 10^5^ cells/mL) and incubated overnight. Then, PdPt@HA NPs (20 µg/mL) or DOX@PdPt@HA NPs (containing the equivalent amount of PdPt@HA NPs) were treated to cells for 1 h at 37 °C in the CO_2_ incubator. To investigate CD44-mediated delivery of the NPs, CD44 receptor of 4T1 cells was blocked by pretreatment of free HA (0.5 mg/mL) 1 h before the addition of the NPs. After fixation with 4% paraformaldehyde, the slides were mounted with anti-fade mounting medium containing DAPI. The images were taken with an Eclipse 90i fluorescence microscope (Nikon Corporation, Tokyo, Japan).

### 2.12. In Vivo Anti-Tumor Effect

Animal experiments were conducted based on the protocols approved by the Animal Ethics Committee of Shenyang Pharmaceutical University (protocol number: 211002300055585). Six-week-old male specific pathogen-free BALB/c mice were housed in a 12:12 h light-dark cycle under controlled temperature of 22 ± 0.5 °C and relative humidity of 50–60%. Subcutaneous implantation model was induced by transplanting 2 × 10^6^ of 4T1 cells into the axilla of right forelimb of mice on day 0. On day 7, the tumor-bearing mice were randomly divided into 8 groups that received intravenous injection of 100 μL saline with or without NIR laser irradiation, free DOX (9.32 μg/mL) with or without NIR laser irradiation, PdPt@HA NPs (40 μg/mL) with or without NIR laser irradiation and DOX@PdPt@HA NPs (containing equivalent dosage of DOX and PdPt@HA NPs) with or without NIR laser irradiation (*n* = 6 in each group). Six hours after intravenous injection of saline, DOX, PdPt@HA NPs or DOX@PdPt@HA NPs, the tumors of mice subjected to laser irradiation were irradiated with the 808 nm laser at a power density of 0.9 W/cm^2^ for 10 min. During the laser irradiation, real-time temperature of the tumor was recorded and infrared thermographic maps were obtained by the infrared thermal imaging camera. The tumor volumes and body weight of mice were monitored daily. Mice were sacrificed on day 14 and serum was obtained for analyzing the levels of ALT and AST according to the manufacturer’s instructions (Wanlei Life Science, Shenyang, China). Tumor as well as the representative organs including heart, liver, spleen, lung, kidney and brain were excised and weighed. 

### 2.13. H&E Staining and Immunohistochemistry Staining

After fixing with 4% paraformaldehyde, tumor, heart, liver, spleen, lung, kidney and brain fragments were embedded with paraffin and sectioned. H&E staining was then conducted according to the manufacturer’s protocol (Servicebio, Wuhan, China). Moreover, immunohistochemistry staining for caspase-3 and Ki-67 was performed on tumor sections to determine apoptosis and proliferation of tumor cells, respectively. Images were acquired using an Eclipse Ci microscope (Nikon Corporation, Tokyo, Japan) or a Pannoramic 250 digital scanner (3DHistech, Budapest, Hungary). 

### 2.14. Statistics 

Data were expressed as mean ± SE and the statistical comparisons were analyzed by ANOVA and *P < 0.05, **P < 0.01 and ***P < 0.001 were considered statistically significant.

## 3. Results and Discussion

### 3.1. Preparation and Characterization of DOX@PdPt@HA NPs

**Figure 2:**
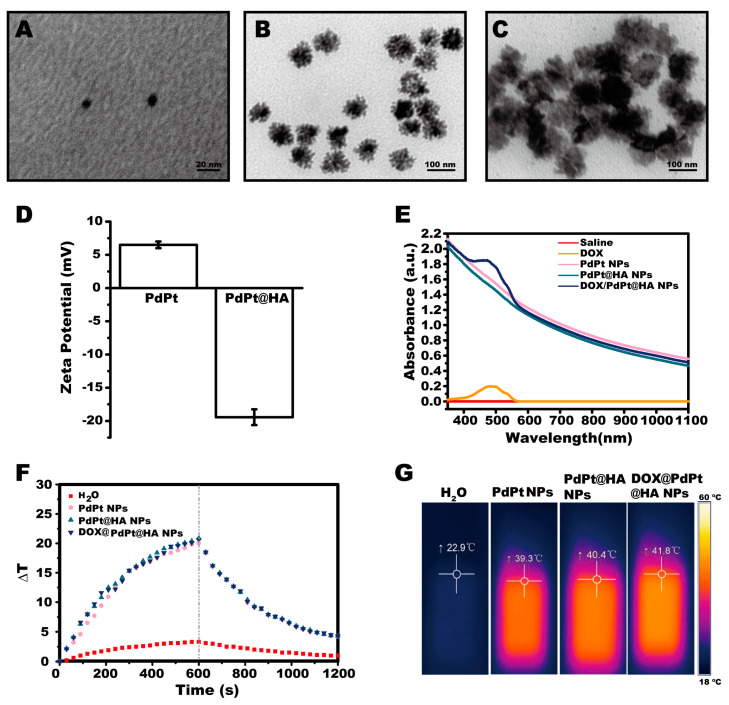
Characterization of DOX@PdPt@HA nanoparticles (NPs). (**A**–**C**) Transmission electron microscopy (TEM) images of the NPs of Pt seed (left), PdPt (middle) and DOX@PdPt@HA (right). (**D**) Evolution of ζ-potential during the process for modification of HA. (**E**) The UV-vis-NIR absorption spectra of DOX as well as PdPt, PdPt@HA and DOX@PdPt@HA NPs in water. (**F,G**) Temperature profiles and representative thermal images of water as well as PdPt, PdPt@HA and DOX@PdPt@HA NPs under the irradiation of a 0.9 W cm^−2^ 808 nm laser for 10 min.

Pt NPs with the size of 8.7 ± 1 nm were used as the seeds to synthesize PdPt NPs (Figure 2A). Consistent with the previous report [21], PdPt NPs exhibited porous wall with numerous dendritic branches on their surface (Figure 2B,C). The mass ratio of Pd to Pt was determined to be 10:34 by elemental analysis of ICP-MS. Thiol functionalized HA (HA-SH) was synthesized and used to cover the surface of PdPt NPs. The decrease of ζ-potential induced by HA-SH coating indicated that the NPs were successfully covered with negatively charged HA (Figure 2D). The sizes of PdPt and DOX@PdPt@HA NPs were 75.3 ± 8.7 nm and 105.2 ± 6.7 nm, respectively. The coating of HA-SH was expected to contribute the increase in the size of DOX@PdPt@HA NPs. Moreover, PdPt and DOX@PdPt@HA NPs were well dispersed in an aqueous solution with PDI values of 0.136 ± 0.017 and 0.143 ± 0.012, respectively.

The FTIR spectroscopy was further employed to determine the structure of DOX@PdPt@HA NPs (Figure S2). The presence of several characteristic bands between 1076 cm^−1^ and 1383 cm^−1^ was associated with the bond of HA and the presence of several bands in the range of 1409–1629 cm^−1^ was associated with the bond of DOX. These results supported the successful synthesis of DOX@PdPt@HA NPs. As shown in UV-vis-NIR absorption spectra, the absorption of PdPt, PdPt@HA and DOX@PdPt@HA NPs was observed in the range of 350–1100 nm. The absorption property suggested that these NPs have potential applications in PTT. Moreover, after the loading of DOX into PdPt NPs, a typical absorption peak at 490 nm for DOX was detected using UV-vis-NIR spectro-photometer, indicating DOX was successfully loaded into the NPs (Figure 2E). The loading content was calculated to be 23.3% by following the Equation (1). In addition, the photothermal properties of the NPs were examined by the infrared thermal imaging camera. Figure 2F,G showed the temperature of the solution of PdPt, PdPt@HA and DOX@PdPt@HA NPs increased ~20 °C under continuous irradiation of 0.9 W cm^−2^ 808 nm laser for 10 min. The results suggested that these NPs could rapidly and efficiently convert the NIR laser energy into heat, arising from a strong photo-absorption in the NIR region. The photothermal conversion efficiency of PdPt, PdPt@HA and DOX@PdPt@HA NPs was further calculated to be 48.6%, 49.8% and 49.1%, respectively (Supplementary Materials, Figure S3). The values are higher than that of the Au nanorods (24%) and CuS NPs (31.1%), which are widely used for PTT [36].

### 3.2. DOX Release Behaviors

**Figure 3:**
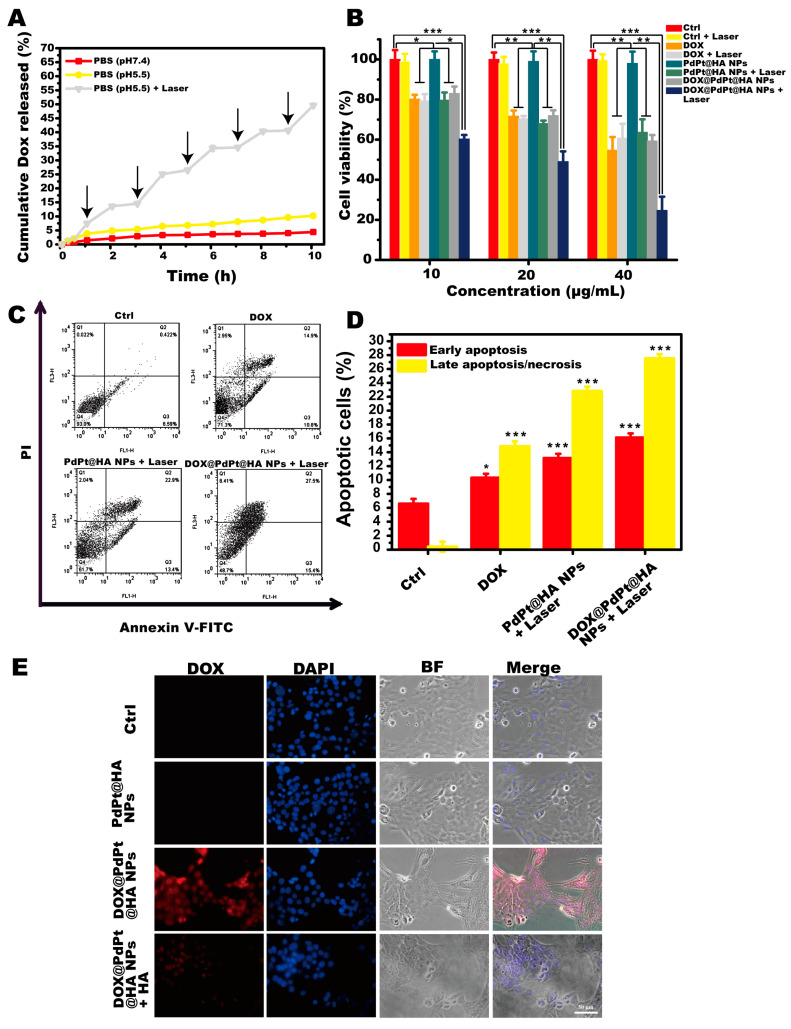
DOX release behaviors and in vitro cytotoxicity of DOX@PdPt@HA NPs. (**A**) NIR laser irradiation triggers release of DOX from DOX@PdPt@HA NPs. (**B**) Cell viability of 4T1 cells treated with different concentration of PdPt@HA NPs, DOX@PdPt@HA NPs or free DOX with or without NIR laser irradiation was determined by 3-(4,5-dimethylthiazol-2-yl)-2,5-diphenyltetrazolium bromide (MTT) assay. (**C**,**D**) Representative flow cytometry scatter plots of Annexin V/PI and quantification of apoptotic ratio of 4T1 cells with different treatments. (**E**) Fluorescence images of 4T1 cells exposed to the NPs with and without pre-incubation of hyaluronic acid (HA) for 1 h. Value represent mean ± SE of 3 independent experiments. *P* values: *P < 0.05, **P < 0.01, ***P < 0.001.

To validate the pH and NIR laser irradiation dual-responsive drug release, the DOX release behaviors of DOX@PdPt@HA NPs in PBS at pH 7.4 and pH 5.5 with or without NIR laser irradiation were investigated. As shown in Figure 3A, the cumulative release rate of DOX from DOX@PdPt@HA NPs was higher at pH 5.5 than those at pH 7.4, suggesting that DOX@PdPt@HA NPs could restrict DOX leakage under physiological conditions but achieve fast drug release in acidic tumor microenvironment. The pH-dependent drug release profile might be linked with the weakened interaction between DOX and PdPt NPs under acidic conditions. In addition, NIR laser irradiation further accelerated the release of DOX at pH 5.5, indicating that DOX release could be remotely controlled by the application of NIR laser irradiation. The NIR laser irradiation-induced increase of temperature could decrease the viscosity of surrounding fluid and promote molecular motion [37]. Therefore, it was reasonable that NIR laser irradiation accelerated DOX release. These results well substantiated the pH and NIR laser irradiation dual-responsive drug release behaviors of DOX@PdPt@HA NPs.

### 3.3. In Vitro Toxicity and Antitumor Efficacy 

MTT assay was performed to detect the cell viability of HFF cells with PdPt or PdPt@HA NPs treatment. The results showed that 10–40 μg/mL PdPt or PdPt@HA NPs neither decreased the cell viability nor induced changes of morphology in nontumorigenic HFF cells, suggesting the negligible toxicity and remarkable biocompatibility of the NPs (Figure S4). To evaluate the in vitro anti-tumor efficacy of the NPs, an MTT assay was conducted to determine the cell viability of 4T1 breast cancer cells incubated with PdPt@HA NPs, DOX@PdPt@HA NPs or free DOX at the equivalent amount with or without NIR laser irradiation. As shown in Figure 3B, DOX or irradiated PdPt@HA NPs effectively decreased cell viability in a dose-dependent manner in comparison with the control group (*P* < 0.05), while NIR illumination or non-irradiation toward PdPt@HA NPs alone did not show obvious effects on cell viability (*P* > 0.05). More importantly, DOX@PdPt@HA NPs presented higher cytotoxicity compared with DOX and irradiated PdPt@HA NPs (*P* < 0.05). 

AV/PI staining was performed to distinguish the viable cells (AV^−^/PI^−^) from dead cells classified as early stage apoptotic (AV^+^/PI^−^) and late stage apoptotic or necrotic (AV^+^/PI^+^) cells [13]. In agreement with previous reports showing that both DOX treatment and PTT were able to trigger DNA damage-induced apoptosis [38,39], our results indicated that DOX or irradiated PdPt@HA NPs significantly increased the percentage of early stage apoptotic and late stage apoptotic or necrotic cells in comparison with the control group (*P* < 0.05). Consistent with the results of the MTT assay, irradiated DOX@PdPt@HA NPs further increased the cell apoptotic rate in both early and late stages compared with that of DOX or irradiated PdPt@HA NPs, suggesting the combination of chemotherapy and PTT exerted a synergistic anti-tumor effect (Figure 3C,D).

CD44-mediated delivery of DOX@PdPt@HA NPs into 4T1 cells was further investigated in vitro. No fluorescence was observed in the cells of the control group or the cells treated with PdPt@HA NPs. In contrast, strong red fluorescence of DOX was detected in 4T1 cells treated with DOX@PdPt@HA NPs, while the pre-incubation of HA effectively decreased the fluorescence intensity (Figure 3E). The results suggested that DOX@PdPt@HA NPs could target tumor cells through CD44-mediated endocytosis. The degradation of HA by intracellular hyaluronidase may further facilitate the release of DOX from the NPs [40].

### 3.4. In Vivo Anti-Tumor Therapy

**Figure 4:**
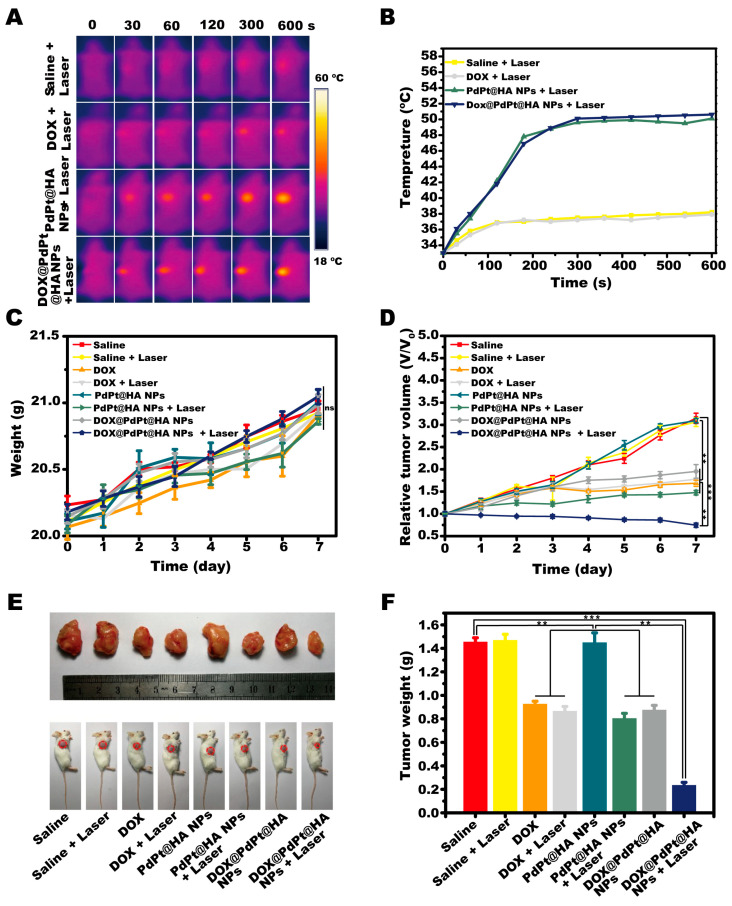
DOX@PdPt@HA NPs for in vivo combination therapy. (**A**) Thermal images of 4T1 tumor-bearing mice recorded by infrared thermal imaging camera. (**B**) Temperature changes of 4T1 tumors in different groups during laser irradiation. (**C**,**D**) Body weight of mice and growth of 4T1 tumors were monitored during the in vivo study. (**E**) Representative photographs of mice and excised tumors at the end of study. (**F**) Weight of tumor collected from animals in different groups. ns, no significant difference. Values represent mean ± SE (*n* = 6 in each group). *P* values: **P < 0.05, **P < 0.01, ***P < 0.001.

The combination effects of chemotherapy and PTT of DOX@PdPt@HA NPs in vivo were evaluated using subcutaneous 4T1 tumor-bearing mice. Temperature changes of tumor were monitored by an infrared thermal imaging camera after mice received therapeutic agents (Figure 4A,B). The results showed that temperature at tumor site in mice with intravenous injection of 40 μg/mL PdPt@HA NPs or DOX@PdPt@HA NPs containing the equivalent amount of PdPt@HA NPs increased ~20 °C after NIR laser irradiation for 10 min, while the irradiation failed to induce obvious increase in the temperature of mice received saline or free DOX injection at equivalent DOX dosage of 9.32 μg/mL. The results demonstrated that both PdPt@HA and DOX@PdPt@HA NPs possessed photothermal effects upon NIR laser irradiation, which was consistent with the results of in vitro study. The temperature increment stimulated by our drug delivery systems in tumor site could be harnessed to kill tumor cells.

During 14-days in vivo study, none of the therapeutic agents induced significant body weight loss (*P* > 0.05), indicating a favorable safety profile of all formulations (Figure 4C). The changes in tumor volume of mice in each group were also measured to evaluate the anti-tumor efficacy after treatment. The tumor size of saline injected-mice with and without NIR laser irradiation increased gradually over time. A significant anti-tumor effect was shown in the group treated with DOX (*P* < 0.01). Under NIR laser irradiation, the injection of PdPt@HA NPs also induced a significant decrease of tumor size of mice (*P* < 0.01), while PdPt@HA NPs treatment without irradiation did not show effect compared with the control group (*P* > 0.05). More importantly, irradiated DOX@PdPt@HA NPs showed a stronger anti-tumor effect than DOX or PdPt@HA NPs with irradiation (*P* < 0.01), indicating a combination effect of chemotherapy of DOX and PTT of PdPt (Figure 4D). Accordingly, the tumor tissues were collected on day 14, the tumor weight of mice with irradiated PdPt@HA NPs and DOX treatment was significantly lower than that of mice in control group (*P* < 0.01) and the irradiated DOX@PdPt@HA NPs further decreased tumor weight (*P* < 0.01) (Figure 4E,F). Thus, the in vivo experiments showed an excellent synergistic effect of DOX@PdPt@HA NPs for chemotherapy and PTT against solid tumors. 

**Figure 5:**
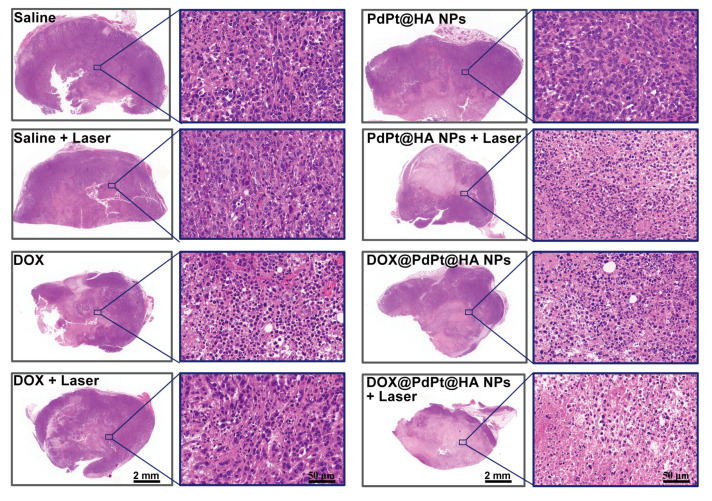
Representative hematoxylin-eosin (H&E) staining images of tumors from animals in different groups. The square in the whole-slide scanned images delineates the magnified subfield on the right.

HA not only acted as gatekeepers of the NPs but also ensured tumor cell targeting by binding with CD44 overexpressed solid tumors [23]. In addition, enzyme-responsive drug release would further occur after the degradation of HA by hyaluronidase abundant in tumor region [23,28,29]. Importantly, the negative charges of NPs after coating with HA-SH could also prolong the blood circulation time due to reduced interactions with blood components [41]. As shown in H&E stained breast cancer histopathology images obtained by whole-slide scanning, dead cells characterized by nuclear fragmentation and nuclei loss were found in the tumor tissue of mice with DOX or irradiated PdPt@HA NPs treatment. And irradiated DOX@PdPt@HA NPs displayed a stronger anti-tumor effect in comparison with chemotherapy or PTT alone. The results indicated HA may assist the localization of the NPs to the tumor sites. Interestingly, a large number of dead cells were found at the central area of tumor in mice with irradiated PdPt@HA or DOX@PdPt@HA NPs treatment, indicating the capability of the NPs to penetrate into solid tumors (Figure 5). 

**Figure 6:**
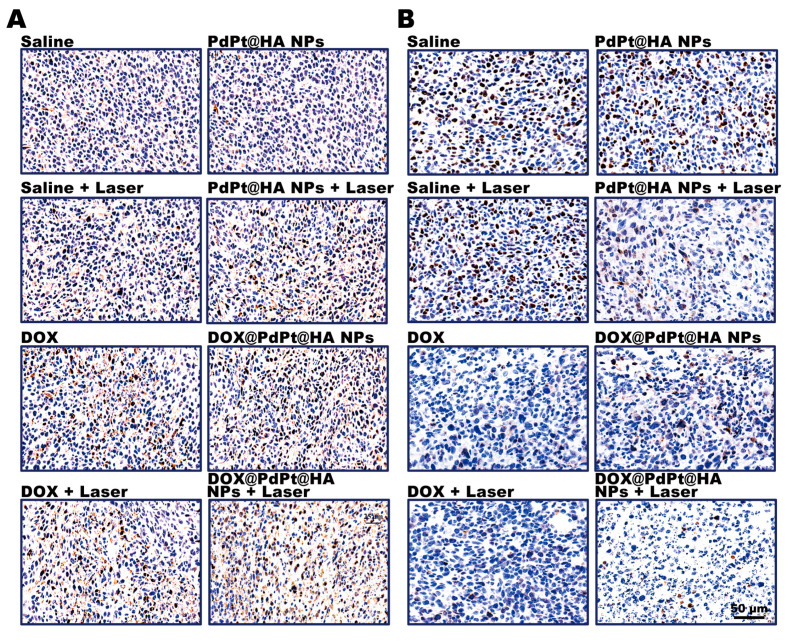
Representative images of caspase-3 (**A**) and Ki-67 (**B**) immunohistochemical staining in tumors from animals with different treatments.

It is well known that the alteration of cancer cell apoptosis and proliferation contributes to the control of cancer development [42]. We further conducted immunohistochemical staining for caspase-3 and Ki-67 to determine the level of apoptosis and proliferation of tumor cells, respectively. Results of immunohistochemical expression of caspase-3 indicated an increased number of apoptotic cells in tumor of mice received DOX or irradiated PdPt@HA NPs treatment. Iradiated DOX@PdPt@HA NPs group showed more caspase-3 positive cells in tumors compared with nonirradiated DOX@PdPt@HA NPs or DOX group (Figure 6A). Moreover, the level of proliferation, determined by Ki-67, was shown to be downregulated by DOX or irradiated PdPt@HA NPs treatment and irradiated DOX@PdPt@HA NPs exerted a stronger inhibitory effect on cell proliferation (Figure 6B). The results indicated the upregulation of apoptosis and downregulation of proliferation contributed to the enhanced therapeutic efficacy of chemo-phototherapy of DOX@PdPt@HA NPs in comparison with chemotherapy or PTT alone. 

**Figure 7:**
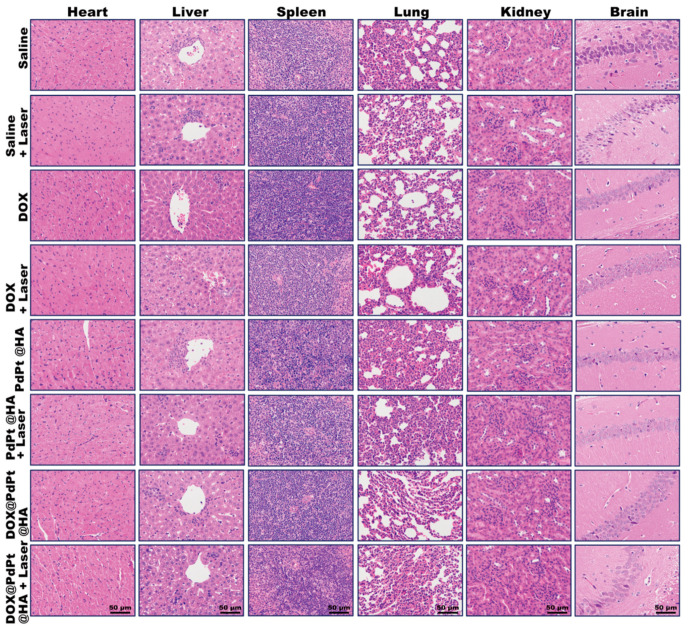
Representative H&E staining images of heart, liver, spleen, lung, kidney and hippocampal CA1 region in brain from animals with different treatments.

A previous study showed that both Pd and Pt NPs did not induce toxicity in aquatic organism, *Daphnia magna* [43]. In our study, the organ coefficient was determined for evaluating the toxicity of PdPt bimetallic NPs on target organs of mice [44,45]. As shown in Table 1, no differences were found in the organ coefficient of heart, liver, spleen, lung, kidney and brain among different groups (*P* > 0.05). Moreover, H&E-stained slices of heart, liver, spleen, lung, kidney and brain displayed no obvious tissue damage occurred in these organs of mice receiving different treatments (Figure 7). The levels of ALT and AST in serum of mice were further determined for the indication of hepatotoxicity [46]. Mice received PdPt@HA or DOX@PdPt@HA NPs injection showed similar levels of ALT and AST in serum compared with the control group (*P* > 0.05), suggesting a favorable safety profile of the PdPt bimetallic NPs. Although DOX treatment alone induced a slight increase of ALT and AST, the alteration was not statistically significant (*P* > 0.05). The results indicated the dosage of DOX used in the current study failed to induce obvious hepatotoxicity (Figure S5), which is accordant with a previous study showing intravenous injection of DOX at less than 15 mg/kg body weight did not significantly affect blood levels of ALT and AST [47].

## 4. Conclusions

In summary, PdPt bimetallic NPs were designed as an NIR stimulus-responsive platform for CD44-targeted and controlled intracellular DOX release. DOX@PdPt@HA NPs were successfully constructed and their ability to conduct chemo-photothermal therapy for breast cancer treatment was determined in the present study both in vitro and in vivo. The prepared DOX@PdPt@HA NPs presented high photothermal conversion efficiency under irradiation of single 808 nm NIR laser. Moreover, DOX@PdPt@HA NPs could respond to the stimuli of NIR illumination to trigger DOX release. Further studies demonstrated that DOX@PdPt@HA NPs exerted a stronger therapeutic efficacy than that of DOX or NIR-irradiated PdPt@HA NPs treatment both in vitro and in vivo, indicating the combined chemo-photothermal therapy with DOX@PdPt@HA NPs exhibits remarkably enhanced therapeutic efficacy in comparison with stand-alone chemotherapeutic or photothermal treatment. In addition, DOX@PdPt@HA NPs induced negligible toxicity in vivo. These results shed light on a promising application of the DOX@PdPt@HA NPs in cancer treatment for their good biocompatibility, CD44-targetability, NIR-responsiveness and chemo-photothermal synergistic effects.

## Figures and Tables

**Table 1:** Effects of different treatments on the organ coefficient of mice.

Groups	Heart	Liver	Spleen	Lung	Kidney	Brain
Ctrl	0.57 ± 0.02	6.15 ± 0.37	2.63 ± 0.08	0.99 ± 0.02	0.74 ± 0.01	2.09 ± 0.03
Ctrl + Laser	0.62 ± 0.04	5.98 ± 0.36	2.68 ± 0.05	0.99 ± 0.04	0.73 ± 0.03	2.15 ± 0.04
DOX	0.53 ± 0.03	5.43 ± 0.3	2.51 ± 0.06	0.98 ± 0.04	0.74 ± 0.06	2.13 ± 0.04
DOX + Laser	0.56 ± 0.04	5.44 ± 0.11	2.70 ± 0.12	1.00 ± 0.04	0.75 ± 0.03	2.20 ± 0.03
PdPt@HA NPs	0.57 ± 0.04	5.51± 0.25	2.63 ± 0.10	0.97 ± 0.05	0.76 ± 0.03	2.16 ± 0.06
PdPt@HA NPs + Laser	0.65 ± 0.07	5.42 ± 0.19	2.66 ± 0.06	1.00 ± 0.04	0.69 ± 0.03	2.18 ± 0.04
DOX@PdPt@HA NPs	0.60 ± 0.03	5.34 ± 0.19	2.62 ± 0.04	0.98 ± 0.06	0.67 ± 0.04	2.15 ± 0.04
DOX@PdPt@HA NPs + Laser	0.58 ± 0.04	5.40 ± 0.02	2.63 ± 0.03	0.96 ± 0.04	0.67 ± 0.02	2.14 ± 0.02

Organ coefficient = organ weight/body weight 100%. Values represent mean ± SE (*n* = 6 in each group). *P* values for all comparisons were > 0.05.

## Supplementary Materials

The following are available online at https://www.mdpi.com/1999-4923/12/7/675/s1, Figure S1: Proton nuclear magnetic resonance (1H-NMR) spectrum of hyaluronic acid (top) and synthesized thiol functionalized hyaluronic acid (bottom), Figure S2: Fourier transform infrared (FTIR) spectra of hyaluronic acid (HA), doxorubicin (DOX), and DOX@PdPt@HA NPs, Figure S3: Determination of the time constant for heat transfer of the system. The sample system time constant (τs) was determined using linear regression of the cooling profile of PdPt NPs (A), PdPt@HA NPs (B) and DOX@PdPt@HA NPs (C), Figure S4: Cell viability of human foreskin fibroblast (HFF) cells incubated with PdPt or PdPt@HA NPs for 24h (A) and cell morphology at indicated time points (B), Figure S5: Levels of alanine aminotransferase (ALT) (A) and aspartate aminotransferase (AST) (B) in serum of mice were quantified for the indication of hepatotoxicity. Values represent mean ± SE (n = 6 in each group).
