# Supplementary material for: NIR Stimulus-Responsive PdPt Bimetallic Nanoparticles for Drug Delivery and Chemo-Photothermal Therapy
Source: Pharmaceutics. 2020 Jul 17;12(7):675. doi: 10.3390/pharmaceutics12070675 (PMC7408621; doi:10.3390/pharmaceutics12070675)
Supplement: Supplementary file 1 [file pharmaceutics-12-00675-s001.pdf]

pharmaceutics

Supplementary Materials: NIR Stimulus-Responsive PdPt Bimetallic Nanoparticles for Drug Delivery and Chemo-Photothermal Therapy

Chun Chu, Zhihong Bao, Meng Sun, Xiaowei Wang, Hongyan Zhang, Weiguo Chen, Yang Sui, Ji Li, Yuanyuan Zhuang and Dongkai Wang *

Supplementary Methods

Determination of photothermal conversion efficiency

The photothermal conversion efficiency (η) of the prepared nanoparticles (NPs) was calculated by using the following equation

$$\eta = \frac{hS(T_{\max}\text{-}T_{\text{surr}})\text{-}Q_{\text{dis}}}{I(1-10^{-A_{808\text{nm}}})} \quad (1)$$

where h is the heat transfer coefficient; S is the irradiated area; I is the laser power density (0.9 W cm^{-2}); A_{808nm} is the absorbance at 808 nm; T_{max} and T_{surr} are the maximum temperature and the surrounding temperature; Q_{dis} is the baseline energy input from the light absorption by the solvent. In Equation (1), hS value is calculated using the following equations:

$$hS = \frac{mC_p}{\tau_s} \quad (2)$$

$$t = -\tau_s \ln\theta \quad (3)$$

$$\theta = \frac{T - T_{surr}}{T_{\max} - T_{surr}} \quad (4)$$

m and C_p are the mass (1.0 g) and the thermal capacity, respectively; T is the temperature at the cooling time (t). By linear fitting cooling time (t) to negative natural logarithm of temperature ($-\ln\theta$), τ_s was determined to be 329.83, 322.80 and 326.68 s for PdPt NPs, PdPt@HA and DOX@PdPt@HA NPs, respectively (Figure S1).

The value of Q_{dis} of water was calculated to be 30.54 mW using the following equation:

$$Q_{dis} = h_0 S(T_{max,\,water} - T_{surr,\,water}) \quad (5)$$

Thus, η of PdPt NPs, PdPt@HA and DOX@PdPt@HA NPs was calculated to be 48.6%, 49.8% and 49.1%, respectively.

Supplementary Results

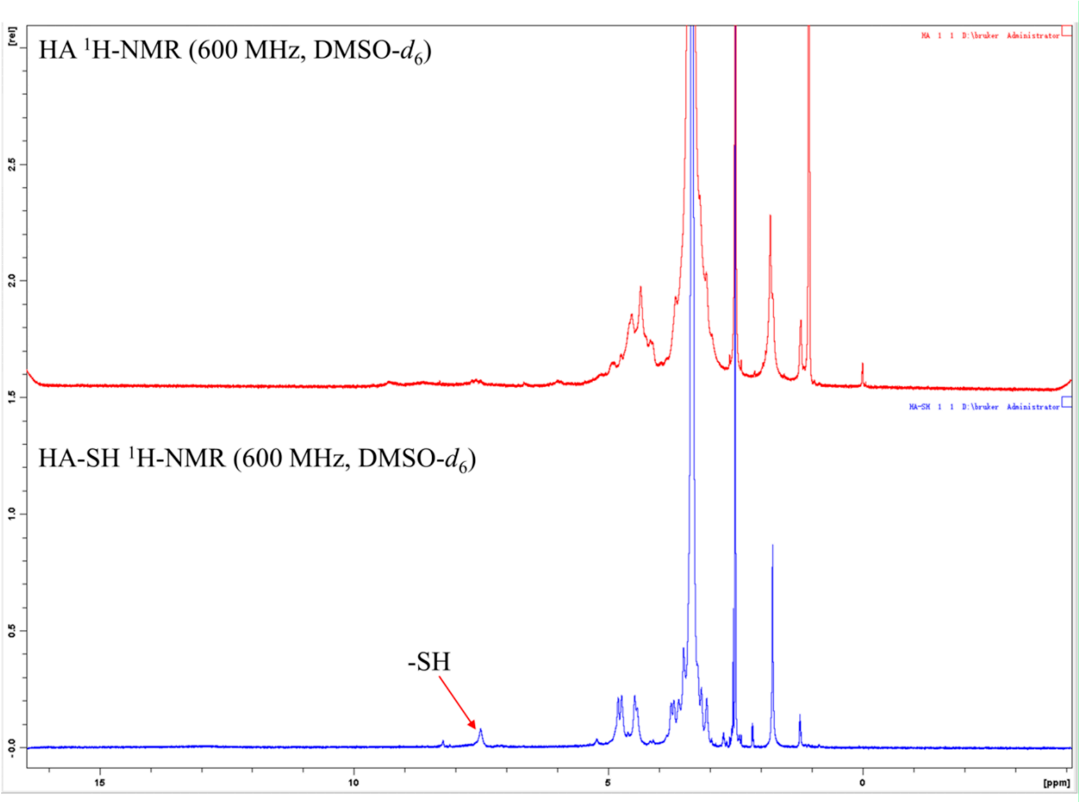

Figure S1. Proton nuclear magnetic resonance (¹H-NMR) spectrum of hyaluronic acid (top) and synthesized thiol functionalized hyaluronic acid (bottom).

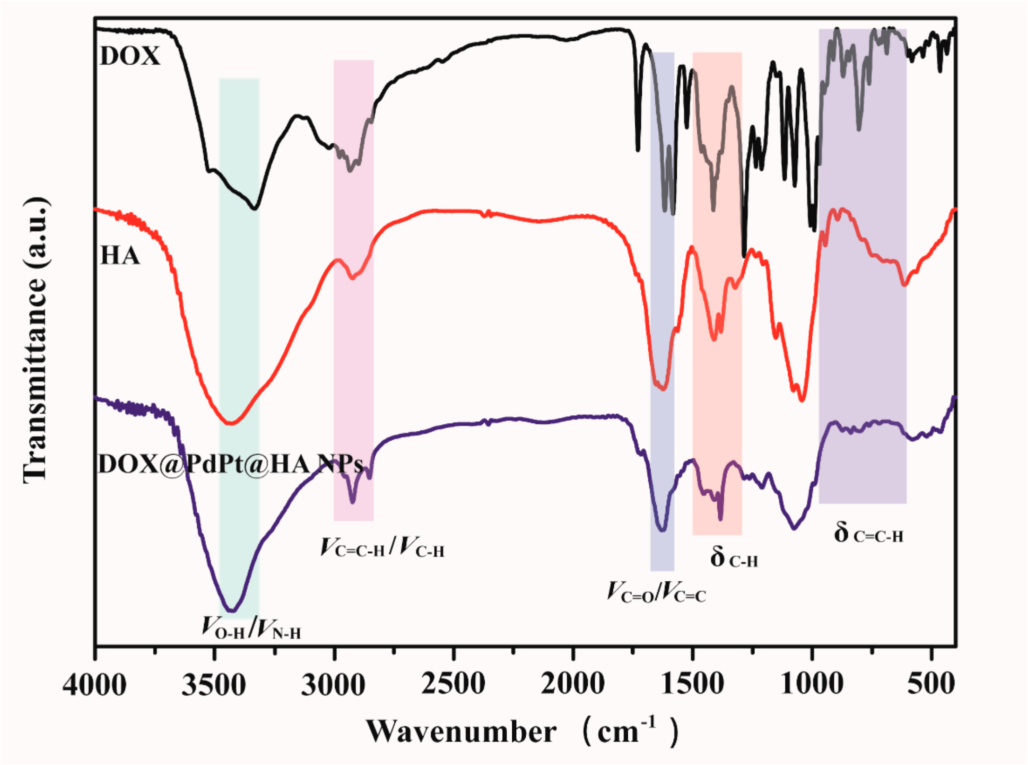

Figure S2. Fourier transform infrared (FTIR) spectra of hyaluronic acid (HA), doxorubicin (DOX), and DOX@PdPt@HA NPs.

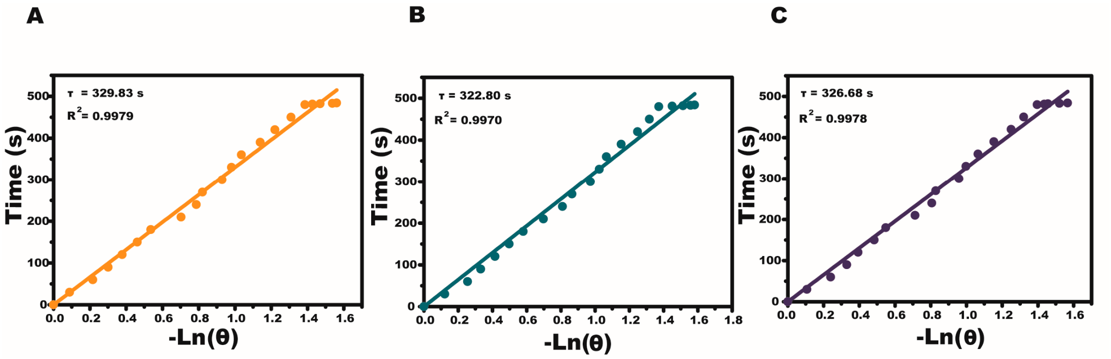

Figure S3. Determination of the time constant for heat transfer of the system. The sample system time constant (τ_s) was determined using linear regression of the cooling profile of PdPt NPs (**A**), PdPt@HA NPs (**B**) and DOX@PdPt@HA NPs (**C**).

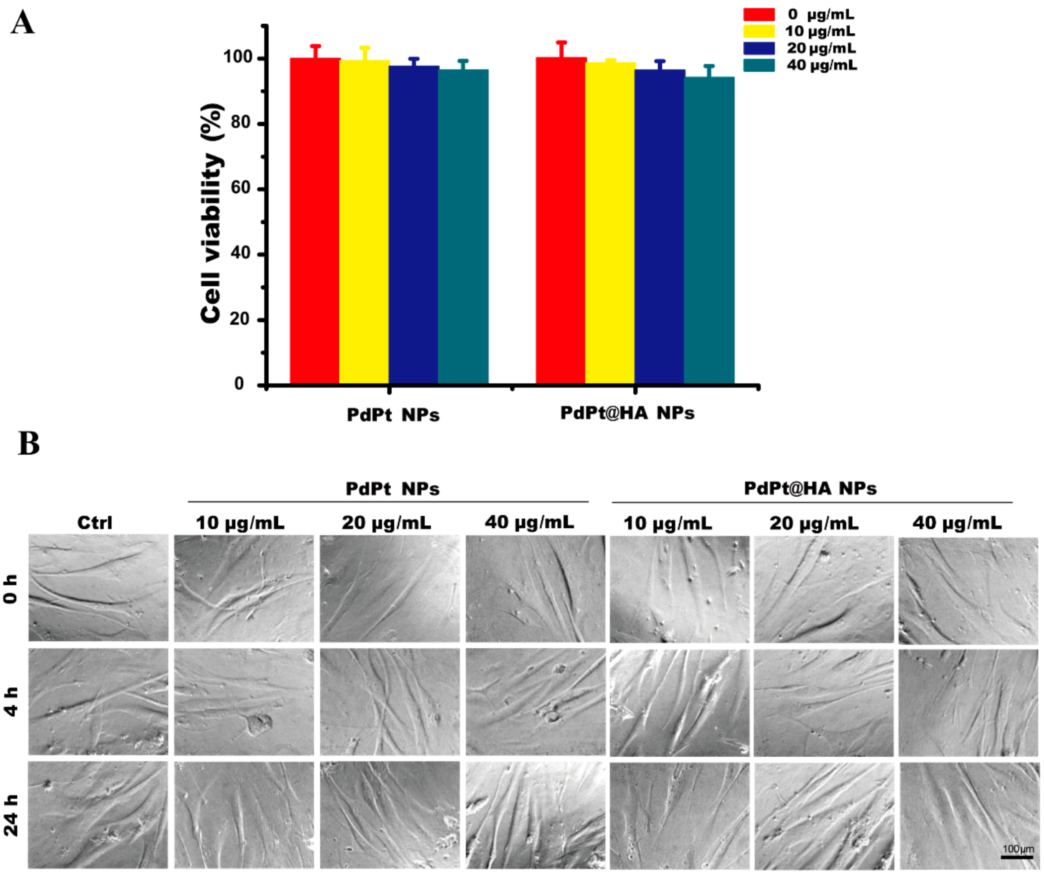

Figure S4. Cell viability of human foreskin fibroblast (HFF) cells incubated with PdPt or PdPt@HA NPs for 24h (**A**) and cell morphology at indicated time points (**B**).

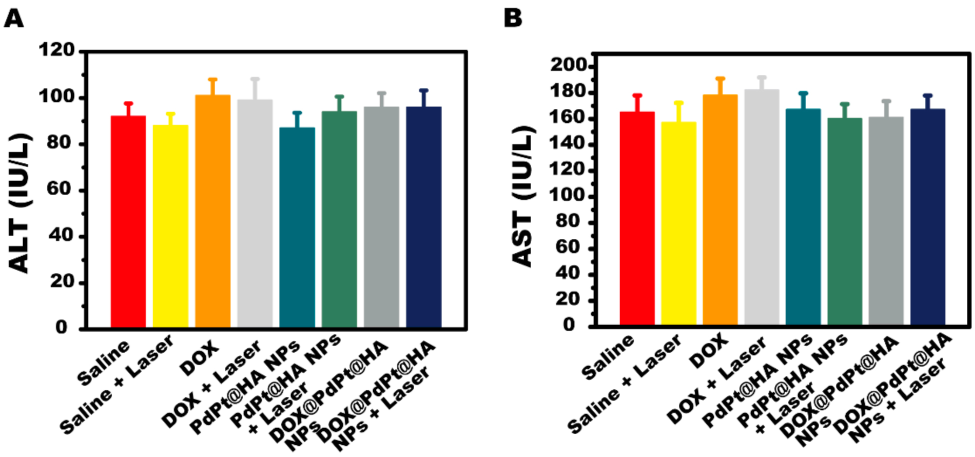

Figure S5. Levels of alanine aminotransferase (ALT) (**A**) and aspartate aminotransferase (AST) (**B**) in serum of mice were quantified for the indication of hepatotoxicity. Values represent mean ± SE (n = 6 in each group).
